# Incomplete Dll4/Notch signaling inhibition promotes functional angiogenesis supporting the growth of skin papillomas

**DOI:** 10.1186/s12885-015-1605-2

**Published:** 2015-08-28

**Authors:** Dusan Djokovic, Alexandre Trindade, Joana Gigante, Mario Pinho, Adrian L. Harris, Antonio Duarte

**Affiliations:** 1 Centro Interdisciplinar de Investigação em Sanidade Animal (CIISA), Universidade de Lisboa (ULisboa), Lisbon, Portugal; 2 Cancer Research UK Molecular Oncology Laboratories, Weatherall Institute of Molecular Medicine, University of Oxford, Oxford, UK

## Abstract

**Background:**

In invasive malignancies, Dll4/Notch signaling inhibition enhances non-functional vessel proliferation and limits tumor growth by reducing its blood perfusion.

**Methods:**

To assess the effects of targeted *Dll4* allelic deletion in the incipient stages of tumor pathogenesis, we chemically induced skin papillomas in wild-type and *Dll4*^+/−^ littermates, and compared tumor growth, their histological features, vascularization and the expression of angiogenesis-related molecules.

**Results:**

We observed that *Dll4* down-regulation promotes productive angiogenesis, although with less mature vessels, in chemically-induced pre-cancerous skin papillomas stimulating their growth. The increase in endothelial activation was associated with an increase in the VEGFR2 to VEGFR1 ratio, which neutralized the tumor-suppressive effect of VEGFR-targeting sorafenib. Thus, in early papillomas, lower levels of Dll4 increase vascularization through raised VEGFR2 levels, enhancing sensitivity to endogenous levels of VEGF, promoting functional angiogenesis and tumor growth.

**Conclusion:**

Tumor promoting effect of low-dosage inhibition needs to be considered when implementing Dll4 targeting therapies.

## Background

Delta-like 4 (Dll4)-mediated Notch signaling critically influences blood vessel formation in both physiological and pathological settings. During embryonic development, this signaling is absolutely required for normal arterial specification [1]. In addition, Dll4/Notch fundamentally participates in the regulation of embryonic [1, 2], post-natal developmental [3–6], regenerative [7] and tumor sprouting angiogenesis [8–14]. It mediates communication between adjacent endothelial cells (ECs) that lead the sprout formation and adjacent ECs that, under Dll4/Notch control, remain in the quiescent state in pre-existing vasculature or rather proliferate then migrate, forming the trunk of the new vessel [5, 6, 13]. Mechanistically, Dll4/Notch enables the selective EC departure from pre-existing activated endothelium and organized sprout outgrowth by decreasing the VEGFR2/VEGFR1 ratio and therefore reducing the sensitivity of signal-receiving ECs to VEGF.

Elevated Dll4 expression predicts poor prognosis in different cancers [14–17]. Previous studies have shown that although Dll4/Notch blockade potentiates the tumor-driven angiogenic response, it inhibits tumor growth due to the formation of immature and poorly functional vessels that result in reduced tumor perfusion [8–13, 18]. Additionally, Dll4/Notch inhibition has been found to reduce the frequency of cancer stem cells [19]. Although these findings indicate that the Dll4/Notch blockade may provide an effective way to improve cancer control, the capacity for normalization of the aberrant vascular network to Dll4/Notch inhibition remains undetermined. Moreover, therapeutic inhibition of Dll4 signaling may face important safety limitations since chronic Dll4/Notch impairment was found to destabilize normal endothelium giving origin to the formation of benign vascular tumors [12, 20, 21]. Nevertheless, several studies with different Dll4 blocking antibodies are now proceeding through phase I trials.

Despite the wealth of information regarding the effects of Dll4/Notch inhibition in invasive neoplasms, little is known regarding its role in benign and early, pre-cancerous lesions. We have previously shown that *Dll4* heterozygote mice can produce functional neo-angiogenesis and improve vascular function in the context of physiological angiogenesis [7]. The present study was undertaken to assess the effects of targeted *Dll4* allelic deletion in the incipient stages of tumor pathogenesis. For this purpose, we used a classic 7,12-dimethylbenz[a]anthracene (DMBA)/12-O-tetradecanoylphorbol-13-acetate (TPA)-induced skin carcinogenesis model wherein the initiating carcinogen, DMBA, results in an activating mutation in the *H*-*ras* gene and generation of “initiated” epidermal cells [22]. These cells form papillomas progressing, in the later phase, to squamous carcinomas and sharing the same *H*-*ras* mutation with some human lesions, like papillomas in Costello syndrome and epidermal nevi [23, 24].

## Methods

### Mice

The CD1 wild-type (WT) and heterozygous *Dll4*^+/−^ mice were generated and housed as previously described [1, 12]. The mice were fed standard laboratory diet and drinking water ad libitum. All animal-involving procedures were approved by the Faculty of Veterinary Medicine of Lisbon Ethics and Animal Welfare Committee (Approval ID: PTDC/CVT/71084/2012).

### Chemically-induced skin tumourigenesis

Male, 8-week old WT and *Dll4*^+/−^ littermates (*n* = 12 for each group) were treated with a single dose of 25 μg of 7,12-dimethylbenz[a]anthracene (DMBA; Sigma, St. Louis, MO) in 200 μL acetone per mouse applied to shaved dorsal skin. Beginning a week after DMBA-induction, tumor onset and growth was promoted by treating mice twice a week for 19 weeks with 4 μg of 12-O-tetradecanoylphorbol-13-acetate (TPA; Sigma, St. Louis, MO) in 100 μL of dimethyl sulfoxide (DMSO) per mouse. The appearance of skin lesions was monitored and recorded weekly. Mouse weight and tumor sizes (diameters) were periodically measured and lesion diameters were converted to tumor volume using the following formula: V = length × width × height × 0.52. Tumor burden of each individual mouse was calculated as the sum of its individual tumour volumes. Twenty weeks after the DMBA initiation, mice were anesthetized by intraperitoneal (i.p.) injection of 2.5 % tribromoethanol (Sigma-Aldrich, St. Louis, MO) and total blood was collected by axillary bleeding from six animals of each genotype for the determination of VEGF, cleaved VEGFR1 and cleaved VEGFR2 concentrations. The remaining WT and Dll4^+/−^ mice (*n* = 6 for each genotype) were perfused with biotin-conjugated lectin (Sigma, St. Louis, MO), as described below, for the assessment of tumor vessel functionality. The skin tumors were then dissected from all WT and Dll4^+/−^ mice and processed for histological or molecular analyses.

### Tumor tissue preparation, histopathology and immunohistochemistry

Skin tumor samples were processed as previously described [12] and cryosectioned at 20 μm. Sections were stained with hematoxylin (FlukaAG Buchs SG, Switzerland) and eosin Y Sigma Chemicals, St. Louis, MO). Double fluorescent immunostaining to platelet endothelial cell adhesion molecule (PECAM) and vascular smooth muscle cell marker alpha smooth muscle actin (α-SMA) was also performed on tumor tissue sections to examine tumor vascular density and vessel maturity. Rat monoclonal anti-mouse PECAM (BD Pharmingen, San Jose, CA) and rabbit polyclonal anti-mouse α-SMA (Abcam, Cambridge, UK) were used as primary antibodies and appropriate species-specific antibodies conjugated with Alexa Fluor 488 and 555 (Invitrogen, Carlsbad, CA) were engaged as secondary antibodies. Nuclei were counterstained with 4′,6- diamidino-2-phenylindole dihydrochloride hydrate (DAPI; Molecular Probes, Eugene, OR). Fluorescent immunostained sections were examined under a Leica DMRA2 fluorescence microscope with Leica HC PL Fluotar 10 and 20X/0.5 NA dry objective, captured using Photometrics CoolSNAP HQ, (Photometrics, Friedland, Denmark), and processed with Metamorph 4.6–5 (Molecular Devices Sunnyvale, CA). Morphometric analyses were performed using the NIH ImageJ 1.37v program. To estimate vessel density, we measured the percentage of tumor stroma surface occupied by a PECAM positive signal. Mural cell recruitment was assessed as a measure of vascular maturity by quantitating the percentage of PECAM-positive structures lined by α- SMA-positive coverage. For the assessment of VEGFR2 expression in papillomas, immunostaining was performed using purified rat anti-mouse VEGFR2 (BD Pharmingen, San Jose, CA) and appropriate secondary antibody conjugated with Alexa Fluor 555 (Invitrogen, Carlsbad, CA). PDGFR-β expression was assessed by double PECAM/PDGFR-β immunostaining for which we used rat monoclonal anti-mouse PECAM (BD Pharmingen, San Jose, CA), rabbit monoclonal anti-mouse PDGFR-β (Cell Signalling Technology, Denver, MA), and appropriate secondary antibodies conjugated with Alexa Fluor 488 and 555 (Invitrogen, Carlsbad, CA). Papillomas from WT and mutant mice were compared upon the measurement of the percentage of tumor stroma surface occupied by a VEGFR2 positive signal as well as the measurement of the percentage of PECAM-positive structures lined by PDGFR-β-positive coverage.

### Vessel perfusion study

To assess vascular perfusion and determine the functional fraction of the tumor circulation, the anesthetized mice were injected via caudal vein with a solution of biotin-conjugated lectin from *Lycopersicon esculentum* (100 μg in 100 μl of PBS; Sigma, St. Louis, MO), which was allowed to circulate for 5 min before transcardially perfusion with 4 % PFA in PBS for 3 min. Tumor samples were collected and processed as described above. Tissue sections (20 μm) were stained with rat monoclonal anti-mouse PECAM antibody (BD Pharmingen, San Jose, CA), followed by Alexa 555 goat anti-rat IgG (Invitrogen, Carlsbad, CA). Biotinylated lectin was visualized with Strepatavidin-Alexa 488 (Invitrogen, Carlsbad, CA). The images were obtained and processed as described above. Tumor perfusion was quantified by determining the percentage of PECAM-positive structures that co-localized with Alexa 488 signal, corresponding to lectin-perfused vessels.

### Serum VEGF, VEGFR1 and VEGFR2 measurement

Blood was allowed to clot during 45 min at 37 °C and then centrifuged during 10 min at 1000 × g. VEGF, VEGFR1 and VEGFR2 serum levels were measured by enzyme-linked immunosorbent assay (ELISA; R&D Systems), as described [25].

### Quantitative transcriptional analysis

Using a SuperScript III FirstStrand Synthesis Supermix qRTPCR (Invitrogen, Carlsbad, CA), first-strand cDNA was synthesized from total RNA previously isolated with RNeasy Mini Kit (Qiagen, Valencia, CA) from skin tumors developed by WT and *Dll4*^+/−^ mice (*n* = 10 tumors for each genotype). Real-time PCR analysis was performed as described [26] using specific primers for *β*-*actin*, *Dll4*, *Hey2*, *PDGF*-*β*, *EphrinB2* and *Tie2*. Gene expression levels were normalized to β-actin. Primer pair sequences are available upon request.

### Sorafenib therapy assay

For the evaluation of combined effect of Dll4 allelic deletion and sorafenib administration, 8-week old WT and Dll4^+/−^ male mice were separated in two equal sub-groups for each genotype (*n* = 4 for each of four experimental sub-groups) and skin tumorigenesis was induced and promoted as described above. Sorafenib was formulated twice a week at 4-fold (4×) concentration in a cremophor EL (Sigma, St. Louis, MO)/ethanol 50:50 solution. The oral solutions were prepared on the day of use by dilution to 1× with cremophor EL/ethanol/water mixture (12.5:12.5:75). Beginning 13 weeks after DMBA induction, a sub-group of WT and a sub-group of Dll4^+/−^ mice was treated by oral gavage for 21 days with sorafenib (40 mg/kg/day) while the mice from the remaining WT and *Dll4*^+/−^ sub-groups received only the vehicle (cremophor EL/ethanol/water 12.5:12.5:75 mixture). Prior and during the treatments, the weight of all mice and their skin tumors were measured once a week and at the experiment endpoint when the mice were sacrificed, tumors dissected and PECAM/α-SMA double immunostaining performed as described above.

### Statistical analyses

The sample size determination was empiric, based on previous experience [11, 12] and the occurrence of DMBA/TPA-induced skin lesions in 100 % of CD1 WT mice. All measurements in the study were independently performed by two technicians who were blind to the group to which the experimental animals belonged. For each measurement, the average of values obtained by two technicians was taken as the measurement result. Data processing was carried out using the Statistical Package for the Social Sciences version 15.0 software (SPSS v. 15.0; Chicago, IL). Statistical analyses were performed using Mann–Whitney-Wilcoxon test. All results are presented as mean ± SEM. *P*-values <0.05 and <0.01 were considered significant (indicated in the figures with *) and highly significant (indicated with **), respectively.

## Results and discussion

### *Dll4* allelic deletion promotes the growth of induced skin papillomas

**Fig. 1:**
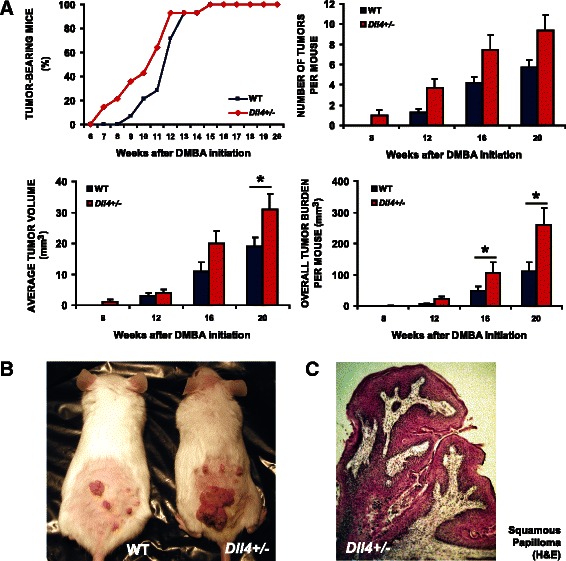
*Dll4* allelic deletion promotes the onset and growth of chemically- induced skin tumors. **a** Tumor kinetics in DMBA/TPA-treated WT and *Dll4*^+/−^ mice. **b** Representative WT and *Dll4*^+/−^ mouse 20 weeks after DMBA-initiation. **c** Hematoxylin and eosin staining of a squamous papilloma sampled from a *Dll4*^+/−^ mouse

To study the Dll4/Notch function in mouse skin tumors and evaluate the effect of its suppression, we monitored skin lesion formation and evolution in WT and *Dll4*^+/−^ mice during 20 weeks. As presented in Fig. 1a, *Dll4*^+/−^ mice started developing skin tumors as early as week 6 after DMBA-initiation and by week 10.5 of the study, 50 % of *Dll4*^+/−^ mice developed at least one lesion (tumor latency). Tumor onset and latency were delayed in WT animals for 2 and 1 week, respectively. Regarding tumor multiplicity, increased number of lesions per mouse was observed in *Dll4*^+/−^ mice compared to WT controls throughout the experiment, but without statistical significance. Importantly, we observed a higher frequency of larger tumors, significantly increased mean lesion volume and overall tumor burden (calculated as the sum of tumor volumes per mouse) in *Dll4*^+/−^ relative to WT mice at the experiment endpoint; *p* < 0.05 (Fig. 1a and b). Body weight of WT and *Dll4*^+/−^ mice did not change significantly at any time during the course of the study, suggesting a low level of systemic carcinogen toxicity.

Histological analysis revealed that the two experimental groups were uniform in terms of tumor histopathology, presenting similar rates of malignant tumor conversion (Table 1). DMBA alone can cause angiomatous lesions. However they were neither observed in WT nor in Dll4+/− mice. With the exception of a single squamous cell carcinoma (SSC), grade I, observed in each group, all remaining skin alterations were exophytic lesions classified as benign squamous papillomas displaying hyperkeratotic epidermal projections with *foci* of dyskeratosis and dysplasia, intact basement membrane and superficial dermal inflammation (Fig. 1c). Thus, reduced Dll4/Notch signaling does not necessarily prevent tumor growth. On the contrary, in this case, in the context of chemically-induced skin papillomas it did promote it. *Dll4* allelic deletion reduced papilloma latency and promoted their multiplicity and growth, although without affecting malignant progression of these lesions.

**Table 1:** Histopathological analysis of lesions from DMBA/TPA-treated wild type and *Dll4*^+/−^ mice

Diagnosis	Wild-type	*Dll4*+/−
Squamous papilloma	68	112
SCC grade I	1	1
Total	69	113

Results shown are from 182 lesions from WT and *Dll4*^+/−^ littermates (*n* = 12 for each group) treated with a single initiation dose of 25 μg DMBA followed by 4 μg TPA twice a week for 19 weeks. *SSC* squamous cell carcinoma

### Impaired Dll4/Notch signaling results in excessive, less mature but productive angiogenic response in induced skin tumors

**Fig. 2:**
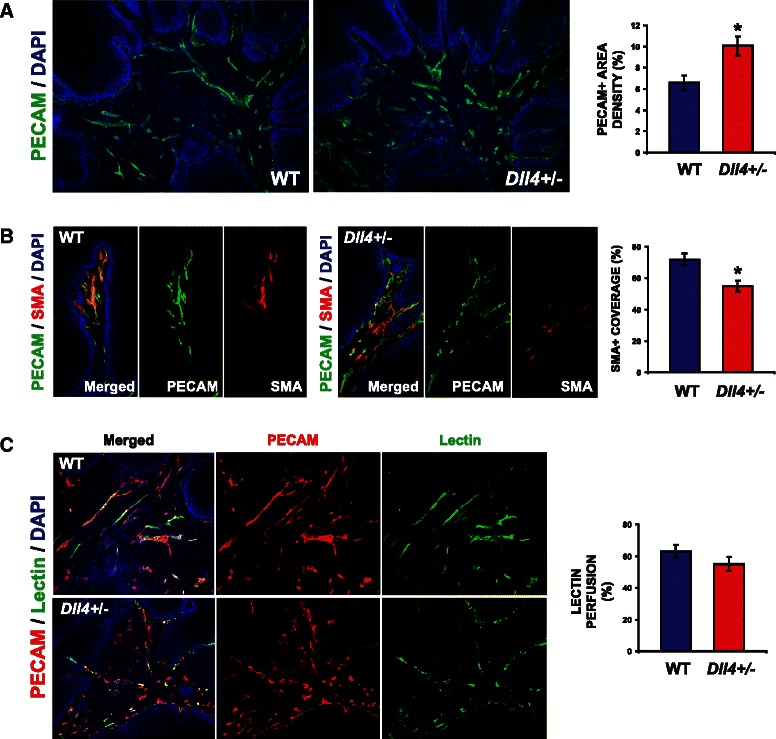
Partial Dll4/Notch inhibition due to haploid *Dll4* deletion enhances less mature but productive angiogenesis in skin papillomas. **a** Vascular response examined by PECAM immunostaining indicating increased sprouting and network disorganization with reduced vessel calibers in *Dll4*^+/−^ vs. WT mice. **b** Mural cell coverage examined by double immunostaining of PECAM and α-SMA showing reduced recruitment of perivascular cells in *Dll4*^+/−^ tumors. **c** Tumor vessel competence evaluated by lectin perfusion and subsequent double staining to PECAM and biotinylated lectin demonstrating a reduction in the fraction of perfused vessels in *Dll4*^+/−^ vs. WT mice

To better understand the observed effect on tumor growth of reduced Dll4/Notch signaling, we examined the vascular morphology of the tumors derived from WT and *Dll4*^+/−^ mice. As presented in Fig. 2a, the WT papillomas were found to be vascularized lesions characterized by irregular vessel formation. However, the WT tumor vasculature presented high degrees of vessel maturity (Fig. 2b) and functionality (Fig. 2c), as indicated by PECAM/α-SMA and PECAM/lectin colocalization, respectively. In comparison, *Dll4*^+/−^ papillomas showed ~35 % increase in vessel density, measured as the PECAM– positive area per tumor stromal surface (*p* < 0.05, Fig. 2a), forming more disorganized endothelial networks with pronounced branching and thin interconnections (data not shown). In addition, α-SMA–positive cells lining PECAM–positive endothelium were significantly reduced (31 % reduction, *p* < 0.05, Fig. 2b), a sign of impaired vessel maturation. However, despite the reduced luminal diameters and impaired vessel wall assembly in *Dll4*^+/−^ papillomas relative to the WT mouse lesions (data not shown), the fraction of lectin-perfused vessels, i.e. functional vessels, was not significantly altered (<10 %, Fig. 2c), which means the mutant mice had more functional tumor vessels in absolute terms, given the increased vessel density they displayed. Thus, the 50 % reduction of Dll4 function allowed competent neovessel formation and is likely to be responsible for the increase in papilloma growth. Our results initially appear contradictory to substantial data showing that the Dll4/Notch blockade inhibits functional angiogenesis, yet in this case a genetic modification that lowers *Dll4* expression enhances productive angiogenesis. With regard to the number of vessels, however, the effects are comparable with the Dll4/Notch inhibition in invasive tumors, as *Dll4* heterozygozity also increased vessel density in our experiments. The difference relates to the functionality of the newly-formed vessels.

### *Dll4* down-regulation increases VEGFR function in the skin papillomas

**Fig. 3:**
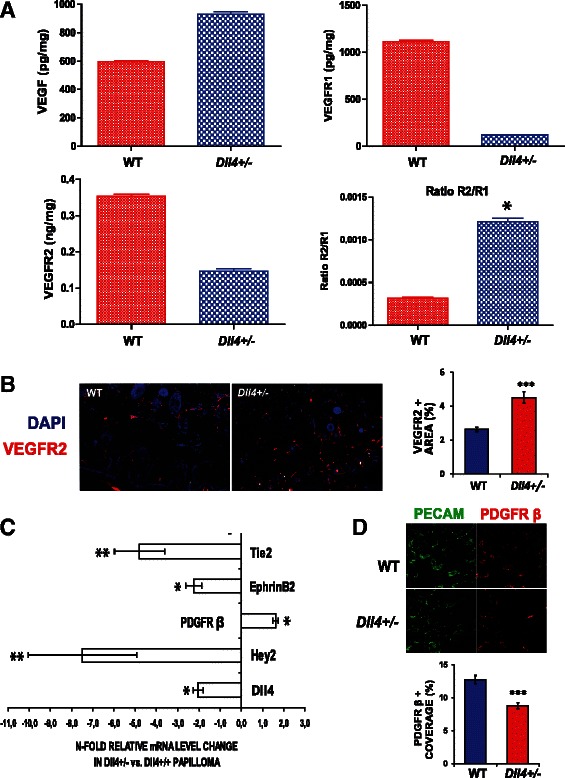
*Dll4* allele deletion affects VEGF/VEGFR signaling and the regulators of vascular smooth muscle cell recruitment. **a** Average serum level of VEGF, VEGFR1, VEGFR2 measured by ELISA and VEGFR2/VEGFR1 (R2/R1) ratio in WT and *Dll4*^+/−^ mice. Between two experimental groups, only VEGFR2/VEGFR1 ratio (R2/R1) differs with statistical significance. **b** VEGFR2 immunostaining showing increased expression of this VEGF-A receptor in *Dll4*^+/−^ vs. control tumors. **c** Differential gene expression in WT vs. *Dll4*^+/−^ papillomas determined by quantitative RT-PCR analyses. **d** Double PECAM/PDGFR-β immunostaining showing reduced PDGFR-β+ vascular coverage and indicating reduced *Pdgfr*-*β* expression in the vasculature of *Dll4*^+/−^ vs. WT papillomas

We propose that the different effects of reduced Dll4 expression on vascular functionality in early vs. late tumors are determined by the background level of VEGF and VEGF/VEGFR signaling. *Dll4* heterozygozity was expected to reduce *Vegfr1* expression and increase *Vegfr2* expression [6], which could account for the increase in angiogenesis and growth of the *Dll4*^+/−^ tumors. We assessed the levels of expression by measurement of cleaved VEGFR1 and VEGFR2 in the serum collected at the end of the experiment, when the tumor bulk was maximal. We detected a marked and statistically significant increase in the serum VEGFR2/VEGFR1 ratio in the *Dll4*^+/−^ mice (Fig. 3a), implying increased endothelial sensitivity to VEGF and enhanced VEGF signaling in *Dll4*^+/−^ papillomas. We also investigated VEGF serum levels but found no difference in the two groups of animals. Nevertheless, with papilloma VEGFR2 immunostaining, we demonstrated, as expected, its highly significant increase in the vasculature of *Dll4*^+/−^ vs. WT lesions (Fig. 3b).

In the context of VEGF levels, which are increased in papillomas, but not as much as in invasive tumors [27], normal Dll4/Notch signaling levels act as a suppressor of VEGF signaling and even a 50 % decrease can result in a change of VEGFR2/VEGFR1 ratio and marked increase in responsiveness to VEGF levels. However, in the context of very high levels of VEGF, Dll4/Notch signaling is likely to be essential to prevent excessive proliferation, aberrant vessel structure and leakiness. So in this case unrestrained VEGF function is detrimental to the functionality of the tumor vascular network. The effects of Angiopoietin-1 (Ang-1) and Angiopoietin-2 (Ang-2) interacting on the Tie2 receptor share some similarity with our findings. Antagonism of Ang-1 by Ang-2 dissociates perivascular cell coverage, and then in the absence of VEGF vessels regress, but in the presence of VEGF, endothelium becomes activated and vessels proliferate [28]. The level of VEGF is critical for the effect of the antagonistic pathway. Similarly, Dll4/Notch blockade enhances EC activation and allows a large number of small vessels to grow, but against the background of very high VEGF levels in invasive tumors these fail to become productive while in the background of lower VEGF levels in benign/early lesions they become functional.

### *Dll4* deletion affects the expression of factors regulating perivascular cell recruitment in chemically-induced skin papillomas

Although *Dll4* haploinsufficiency was found to promote productive angiogenesis in this model, it did indeed negatively influence perivascular cell recruitment to the proliferating tumor capillaries. Thereby, we next used qRT-PCR to analyze WT and *Dll4*^+/−^ papillomas for differential expression of genes known to be involved in EC/ vascular smooth muscle cell interactions (Fig. 3c). We observed that *Dll4*^+/−^ tumors had ~2-fold lower *Dll4* mRNA levels and down-regulated Hey2 expression in comparison to WT, confirming the Notch pathway suppression. Both the angiopoietin receptor Tie2 [29, 30] and EphrinB2 [31] were downregulated upon *Dll4* allelic deletion, which could account for impaired mural cell recruitment in the *Dll4*^+/−^ papillomas. Since the observation of increased *Pdgfr*-*β* mRNA levels was counterintuitive, we performed PDGFR-β immunostaining. In contrast to the whole tumor PDGFR-β increase, we documented its down-regulation in *Dll4*^+/−^ vessels, i.e. reduced PDGFR-β+ coverage of PECAM+ structures in *Dll4*^+/−^ vs. WT mice (Fig. 3d), which is in accordance with reduced perivascular cell recruitment in mutant mouse papillomas.

### Partially inhibited Dll4/Notch signaling decreases the tumor suppressive effect of sorafenib on chemically-induced skin tumors

**Fig. 4:**
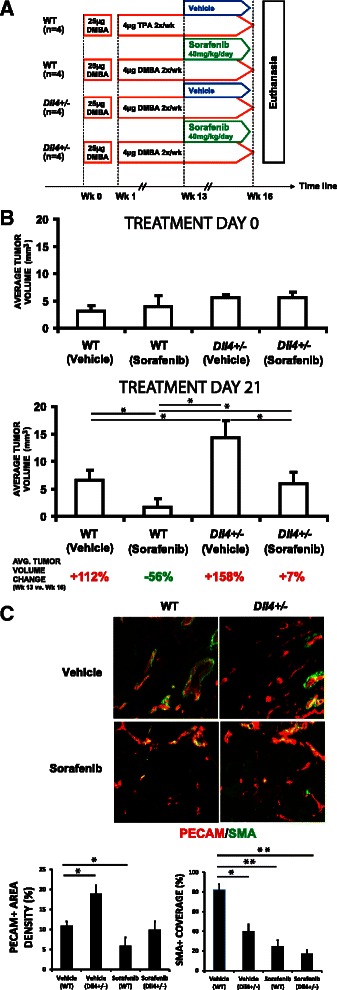
*Dll4* deletion reduces sorafenib efficacy against DMBA/TPA- mediated skin papillomas. **a** Treatment schematic diagram. **b** Tumor volume changes in WT and *Dll4*^+/−^ mice during the treatment with vehicle or sorafenib. **c** Vascular response examined by double PECAM/α-SMA immunostaining indicating reduced sprouting and recruitment of perivascular cells in sorafenib-treated vs. vehicle-treated WT mice, as well as enhanced endothelial proliferation, however, with increasingly impaired vessel wall assembly in sorafenib-treated *Dll4*^+/−^ vs. WT mice

Considering that *Dll4* allele deletion changes the VEGFR function in papillomas, we finally examined the influence of Dll4 downregulation on the efficacy of sorafenib, an oral small-molecule-receptor tyrosine kinase inhibitor which targets include the VEGF receptors [32]. Sorafenib acts either directly on the tumors by inhibiting Raf and Kit signaling, and/or indirectly by suppressing tumor angiogenesis through the inhibition of VEGFR and PDGFR signaling [33] (BAY 43–9006, Nexavar), a dual-action inhibitor that targets RAF/MEK/ERK pathway in tumor cells and tyrosine kinases VEGFR/PDGFR in tumor vasculature). Beginning at week 13 after the DMBA-initiation, when all mice had developed at least one skin lesion and approached the exponential tumor-growth phase, we established 4 experimental groups: *Dll4*^+/−^ treated and control, WT treated and control (Fig. 4a). Control animals received cremophor EL/ethanol/water 12.5:12.5:75 mixture, the sorafenib vehicle, and the treated received sorafenib at 40 mg/kg/day for 3 weeks. Comparing initial and final tumor volumes (Fig. 4b), we observed that vehicle-treated WT tumors showed a 2.1-fold volume increase over the period of 3 weeks while vehicle-treated *Dll4*^+/−^ papillomas presented a more pronounced 2.6-fold volume expansion (*p* <0.05). On the other hand, sorafenib treatment effectively contained tumor growth in WT animals, causing an average regression of 56 % (*p* < 0.05) of tumor volume, after the 3 week administration period. In strike contrast, *Dll4*^+/−^ skin lesions continued to progress despite the sorafenib treatment, although the drug application provided a marked tumor growth retardation compared to vehicle-treated *Dll4*^+/−^ papillomas. Interestingly, vehicle treated WT and sorafenib-treated *Dll4*^+/−^ mice presented very comparable tumor volumes at the treatment endpoint, indicating that 50 % Dll4/Notch inhibition virtually neutralized the effects of sorafenib.

Upon Dll4/Notch inhibition, the papillomas became more sensitive to VEGF due to increased VEGFR function and less responsive to therapeutic VEGFR inhibition. In comparison with sorafenib-treated WT mice, *Dll4*^+/−^ mice treated with this drug presented increased papilloma vascular density, while combined *Dll4* allele deletion and sorafenib application enhanced inappropriate perivascular cell recruitment (Fig. 4c). Decreased efficacy of tyrosine kinases inhibitors might also occur and be even more pronounced in invasive lesions with high VEGF levels. However, the effect on the efficacy of VEGF inhibitors is likely to change with the degree of Dll4/Notch inhibition. More pronounced Dll4/Notch suppression than that observed in *Dll4*^+/−^ mice, might increase their efficacy since the abolishment of Dll4 function promotes unproductive vascular response while concomitant VEGF/VEGFR2 inhibition will reduce the rate of endothelial proliferation. Such an outcome was previously observed with combinational blockade of Dll4/Notch signaling, reducing vascular competence, and EphrinB2 signaling, reducing endothelial proliferation [12], presumably by interfering with the VEGFR trafficking [34].

## Conclusions

The role of Dll4 differs in early and late tumor development. In early papillomas, lower levels of Dll4 increase vascularization through change in VEGFR2 levels and consequently enhance sensitivity to endogenous levels of VEGF. In large invasive cancers that produce greater concentrations of VEGF, downregulation of VEGFR2 by Dll4/Notch signaling is critical to maintain some degree of normal vascular function and organization, and therefore a loss of this buffering mechanism results in excessive vessel sprouting with overall loss of vascular function and tumor perfusion. These observations may be relevant to patients who go onto long term anti-Dll4 therapy, which may be used chronically as is anti-VEGF therapy.

## Abbreviations

Ang-1: Angiopoietin-1
Ang-2: Angiopoietin-2
Dll4: Delta-like 4 ligand
DMBA: 7,12-dimethylbenz[a]anthracene
DMSO: Dimethyl sulfoxide
EC(s): Endothelial cell(s)
PECAM: Platelet endothelial cell adhesion molecule
SMA: Smooth muscle actin
TPA: 12-O-tetradecanoylphorbol-13-acetate
VEGF: Vascular endothelial growth factor
VEGFR1: Vascular endothelial growth factor receptor 1
VEGFR2: Vascular endothelial growth factor receptor 2
WT: Wild-type
